# Laparoscopy in the coronavirus disease 2019 (COVID-19) era

**DOI:** 10.1186/s10397-020-01070-7

**Published:** 2020-05-14

**Authors:** Stefano Angioni

**Affiliations:** Department of Surgical Sciences, Division of Gynecology and Obstetrics, University of Cagliari, Monserrato, Italy

**Keywords:** COVID-19, SARS-CoV-2, Minimally invasive surgery, Laparoscopy, Viral diffusion

## Abstract

The novel severe acute respiratory syndrome coronavirus 2 (SARS-CoV-2) that emerged in China at the end of 2019 has become a pandemic infection that has now involved 200 countries with 465,915 confirmed cases and 21,031 confirmed deaths. Unfortunately, many data have shown that the high number of undocumented infections could have a major role in the rapid diffusion of the disease. In most of the nations involved, non-urgent, non-cancer procedures have been stopped to reallocate medical and paramedical staff to face the emergency. Moreover, concerns have been raised that minimally invasive surgery could be a procedure that carries the risk of virus diffusion in the operating theater during surgery. This paper reports clinical recommendations and scientific studies to assist clinicians in this field.

## Introduction

Minimally invasive surgery and laparoscopy in particular represent the conventional approach to most abdominal and pelvic surgery [1, 2]. The popularity of these techniques is due to many documented advantages, such as short hospitalization, rapid recovery after surgery, higher precision of the surgical maneuvers, and less bleeding [3]. Most surgeries for benign gynecological diseases are performed with laparoscopy [4], and its advantages have increased its application in malignancies [5, 6]. Even less invasive approaches have been developed in recent years, such as the use of very thin instruments in mini- and micro-laparoscopy and the development of single-port access laparoscopy (SPAL) [7, 8]. These evolutions that minimize the port size in the case of mini-laparoscopy or reduce their number by using only one entrance, as in SPAL or transvaginal natural orifice transluminal endoscopic surgery (vNOTES), could be even less invasive than conventional multiport laparoscopy [9, 10]. Nevertheless, everything could change. Indeed, we are facing a new respiratory virus that is modifying our operating room activity. The novel severe acute respiratory syndrome coronavirus 2 (SARS-Cov-2) that emerged in China at the end of 2019 has spread to a pandemic infection in just a few months. It has now involved 200 countries with 465,915 confirmed cases and 21,031 confirmed deaths (data as at March 26, 2020) [11]. Unfortunately, some reports have shown that the high number of undocumented infections could have a major role in the rapid diffusion of this disease [12]. In most of the nations involved, non-urgent, non-cancer procedures have been stopped to reallocate medical and paramedical staff to face the emergency [13]. Moreover, concerns have arisen about the possibility that minimally invasive surgery could be a risky procedure in increasing the virus diffusion in the operating theater during surgery. This paper reports clinical recommendations and published scientific data to help clinicians in this field.

## Laparoscopic surgery and operating theater contamination

Only a few reports in the literature relate to the possible risk to the surgical team of inhalation of viruses from patients during a laparoscopy. In 1996, Des Coteaux et al. demonstrated the presence of breathable aerosols and cell-size fragments in the cautery smoke produced during laparoscopic procedures. The particle sizes ranged from 0.1 to 25 μm [14]. The particle size may depend on the device used [15]. An aerosol is defined as a suspension system of solid or liquid particles in a gas. An aerosol includes both the particles and the suspending gas, which is usually air, and in the case of laparoscopy, CO_2_. Other studies have shown that whole cells can be carried as aerosols in the pneumoperitoneum during laparoscopy in the smoke produced by cauterization [16, 17]. It seems that increasing pneumoperitoneum pressure is correlated to the number of cells found [18]. On the contrary, analysis of the theoretical risk that pneumoperitoneum gas could carry bacteria in aerosol form and spread infection throughout the peritoneal cavity during laparoscopy for infective conditions such as appendicitis was not confirmed in another study, as the pneumoperitoneum gas collected at the end of the procedure did not show any bacterial contamination [19]. Nevertheless, the hepatitis B virus and human papillomavirus DNA have been detected in surgical smoke, although no data exist on surgical team contamination [20, 21]. During open surgery, electrical or ultrasonic cauterization is able to produce aerosols, but some evidence suggests that particle concentrations in smoke seem higher in laparoscopic surgery [22]. The problem of contamination of operating rooms by aerosol is particularly important in relation to the evacuation of the pneumoperitoneum during laparoscopic surgery [23].

## Recommendations

Even if it is still unknown whether SARS-CoV-2 shares the properties of other viruses that can be found in laparoscopic surgical smoke, many scientific societies have published online their recommendations on laparoscopy during this pandemic. The Society of American Gastrointestinal and Endoscopic Surgeons (SAGE) recommends stopping elective surgeries. In urgent or necessary surgeries, since laparoscopy could potentially release viruses, SAGE states that the use of devices to filter released CO_2_ for aerosolized particles, the reduction of medical staff to the minimum inside the operating room, and the use of personal protective equipment (PPE) should be strongly considered [24]. The European Society for Gynecological Endoscopy (ESGE) has also suggested postponing elective surgery for benign conditions until the pandemic ends. The screening of patients for coronavirus infection before planned surgical treatment or the postponement of surgery on suspected or documented SARS-CoV-2-positive patients until their full recovery, if there is no immediate life-threatening situation, is strongly recommended. If this is not possible, surgery must be performed with full PPE for the entire theater staff. Surgery for gynecological cancer should continue unless alternative interim options are possible after the end of the outbreak. The ESGE also provides suggestions to reduce CO_2_ release: (a) closing the port taps before insertion, (b) attaching a CO_2_ filter to one of the ports for smoke evacuation if needed, (c) not opening the tap of any ports unless they are attached to a CO_2_ filter or being used to deliver the gas, (d) reducing the introduction and removal of instruments through the ports, (e) deflating the abdomen with a suction device before removing the specimen bag from the abdomen, (f) deflating the abdomen with a suction device and via the port with a CO_2_ filter at the end of the procedure, and (g) minimizing the use of cauterization [25]. The Royal College of Obstetrics and Gynecology (RCOG) together with the British Society for Gynecological Endoscopy (BSGE) provides similar advice on CO_2_ evacuation and prevention of aerosol transmission and in addition suggests performing laparotomies or deferring operations that have a risk of bowel involvement due to an increased theoretical risk in such cases [26]. The American Association of Gynecologic Laparoscopists (AAGL), along with many other surgical and women’s health professional societies, supports suspension of non-essential surgical care during the immediate phases of the coronavirus disease 2019 (COVID-19) pandemic [27]. In addition to suggestions to reduce aerosol diffusion during and immediately after laparoscopy, the AAGL provides similar advice on screening patients before surgery and suggests additional imaging evaluation (chest computed tomography) prior to any surgical procedure, based on published data on its high predictive ability for early disease [28].

## Conclusions

Our knowledge of this new virus is still very limited. Consequently, the possible risks for health professionals and the risks from operating on an asymptomatic patient positive for SARS-CoV-2 are still unclear. Certainly, in this period, the surgical indications and accurate patient selections should be thoroughly discussed in each case, since it is mandatory to reallocate medical and paramedical staff to face the emergency. Another important issue is to decrease operating room use in order to increase the number of lung ventilators available for the great number of coronavirus patients that need respiratory assistance. The need to limit virus diffusion and the published data on other viruses in surgical smoke, in particular in laparoscopy, should be taken into strong consideration. The ideal situation would be to screen all patients before surgery. If this is not possible, PPE should be used and all the strategies to decrease aerosol diffusion in the operating theater should be followed. I strongly suggest using a device that has a close circuit to maintain the pneumoperitoneum to facilitate smoke evacuation and filtration with a 0.01 μm ultra-low particulate air filter. Another possible suggestion is to use very low CO_2_ pressures. This goal can even be obtained using a deep neuromuscular block to optimize surgical space conditions during laparoscopic surgery at very low insufflation pressure [29]. These strategies increase the cost of the surgery but could improve safety.

## Data Availability

1. World Health Organization. Cornavirus disease (COVID-2019) situation reports 26 March 2020. https://www.who.int/emergencies/diseases/novel-coronavirus-2019 2. SAGES - Society of American Gastrointestinal and Endoscopic Surgeons Recommendations Surgical Response to COVID 19. 2020 https://www.sages.org/recommendations-surgical-response-covid-19/ 3. ESGE Recommendations on Gynaecological Laparoscopic Surgery during Covid-19 Outbreak.2020. https://esge.org/wp-content/uploads/2020/03/Covid19StatementESGE.pdf 4. Joint RCOG/BSGE Statement on Gynaecological laparoscopic procedures and COVID-19. 2020. https://www.bsge.org.uk/news/joint-rcog-bsge-statement-on-gynaecological-laparoscopic-procedures-and-covid-19/ 5. AAGL Joint Statement on Minimally Invasive Gynecologic Surgery during the COVID-19 Pandemic. 2020. https://www.aagl.org/news/covid-19-joint-statement-on-minimally-invasive-gynecologic-surgery/

## Abbreviations

AAGL: American Association of Gynecologic Laparoscopists
BSGE: British Society for Gynecological Endoscopy
CO_2_: Carbon dioxide
COVID-19: Coronavirus disease 2019
DNA: Deoxyribonucleic acid
ESGE: European Society for Gynecological Endoscopy
PPE: Personal protective equipment
RCOG: Royal College of Obstetrics and Gynecology
SAGE: Society of American Gastrointestinal and Endoscopic Surgeons
SARS-CoV-2: Severe acute respiratory syndrome coronavirus 2
SPAL: Single-port access laparoscopy
vNOTES: Transvaginal natural orifice transluminal endoscopic surgery
